# *Pseudomonas psychrophila* Biofilm Formation Inhibition by Thymol Adaptation

**DOI:** 10.1021/acs.jafc.5c09527

**Published:** 2026-01-07

**Authors:** Natacha Caballero Gómez, Julia Manetsberger, Carlos Terriente-Palacios, José G. Vallarino, Nabil Benomar, Hikmate Abriouel

**Affiliations:** † Area of Microbiology, Department of Health Sciences, Faculty of Health Sciences, 16747 University of Jaén, Jaén 23071, Spain; ‡ Department of Molecular Biology and Biochemistry, Institute of Subtropical and Mediterranean Horticulture ‘La Mayora’, University of Malaga - Consejo Superior de Investigaciones Científicas (IHSM-UMA-CSIC), Malaga 29010, Spain

**Keywords:** thymol, biofilms, virulence factors, metabolomic analysis, Pseudomonas psychrophila

## Abstract

This study investigated the effects of thymol (TH) adaptation on biofilm formation and the metabolic profile of the multiresistant slaughterhouse isolate *Pseudomonas psychrophila* M33T02.2. After exposure to increasing sublethal concentrations of thymol, the adapted *P. psychrophila* M33T02.2 showed decreased biofilm-forming capacity, reduced swarming motility, and lower rhamnolipid production compared to the wild-type strain. Confocal microscopy further showed that the biofilms developed by the adapted strain were less homogeneous, confirming their inability to develop well-structured biofilms. To further understand these changes at the metabolic level, high-performance liquid chromatography (UHPLC-Orbitrap-MS/MS) identified redox metabolism intermediates and energy balance-related metabolites as most important variables. 20 metabolites were underexpressed for the TH-adapted strain, including glutathione disulfide, guanosine diphosphate, and flavin adenine mono- and di-nucleotide, among others. Therefore, we conclude that repeated exposure to TH prevents the emergence of resistance mechanisms associated with biofilm formation, acting at the level of redox state and energy imbalance.

## Graphical abstract

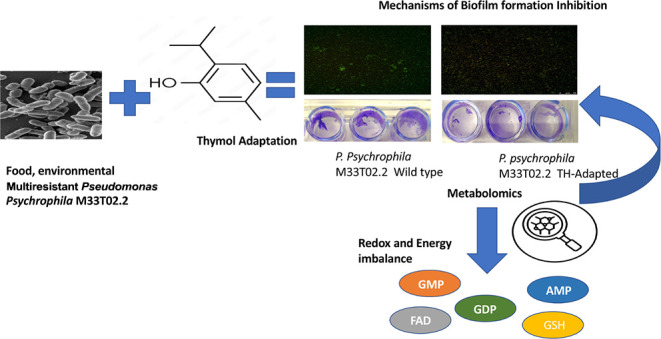

## 1 Introduction

*Pseudomonas* sp. has a remarkable ability to adapt to different environmental situations, allowing for its prolonged survival on abiotic surfaces. This adaptability plays a crucial role in the successful colonization of healthcare and food facilities. Recent evidence has highlighted that nonpathogenic pseudomonads are associated with several human diseases but are still poorly understood in comparison with pathogenic strains such as *P. aeruginosa*.^1^ In this regard, *Pseudomonas* spp. have been widely studied for food spoilage effects; however, given that they are opportunistic pathogens, multiple antibiotic-resistant *Pseudomonas* spp. strains can also pose serious risks in acute and chronic infections.^2^

Bacteria can live as planktonic cells exploring aqueous environments or as a sessile biofilm community. These two bacterial lifestyles differ in terms of virulence factor production and infection strategies,^3^ highlighting that the formation of a biofilm correlates with deep-rooted chronic infections and resistance to both phagocytosis and antimicrobial agents.^4^ Therefore, the development of new, effective, and sustainable control strategies is needed. Historically, plants have been considered a significant source of biologically active molecules with the potential to be used as drug candidates to treat a variety of diseases.^5^ The biological characteristics of essential oils derived from medicinal plants include antibacterial properties.^6–9^ In this regard, terpenoids such as thymol (TH) are important constituents of essential oils. These have received FDA approval for use as food additives and are generally accepted as safe for human consumption.^10^ However, little is known about the adaptive response of bacteria to these components after several exposure cycles. This necessitates greater attention to detect the underlying mechanisms adopted by bacteria to resist various drugs or conditions in diverse niches.

Bacterial adaptation to antimicrobials such as essential oil components, defined by Maisonneuve and Gerdes^11^ as the “persister” state, represents a crucial intermediary stage between sensitive and resistant phenotypes. We previously showed that induction (i.e., adaptation by successive exposure to increasing concentrations) using sublethal TH concentrations reversed antibiotic resistance in planktonic cells (Caballero Gómez et al., under review), while other studies demonstrated an antimicrobial effect of TH against Gram-positive and Gram-negative bacteria.^12^ However, little is known regarding the effects of sublethal TH exposure on the biofilm-forming capacity of multiresistant organisms. Hence, in this study, we investigated the effect of TH adaptation on physiological modifications (via metabolic pathways) involved in biofilm formation under subinhibitory concentrations of TH in *P. psychrophila* M33T02.2, a multiresistant and very strong biofilm-producing strain.^12^

## 2 Material and Methods

### 2.1 Bacterial Strains and Culture Conditions

*Pseudomonas psychrophila* M33T02.2, previously isolated from the white room of a slaughterhouse,^13^ was used in this study under standard and TH adaptation conditions using TH minimum inhibitory concentrations (MIC) as previously determined for this strain (150 μg/mL^12^). Induction of *P. psychrophila* M33T02.2 was carried out as previously described by Caballero Gómez et al. (under review) by inoculating an overnight culture (0.5 McFarland turbidity units) with subinhibitory concentrations (1/2 MIC, “Minimum Inhibitory Concentration”) of thymol (Sigma-Aldrich, Spain) in Tryptone Soy Broth (TSB; Scharlab, Barcelona, Spain) in a total volume of 2 mL, followed by 24–48 h of incubation at 25 °C. This inoculation was repeated in fresh TSB, progressively increasing the concentration of thymol until a final concentration of 2500 μg/mL was reached (TH-adapted strain). The TH-adapted or wild-type (noninduced) strains were stored in TSB containing 20% glycerol at −80 °C until further use. Strains were routinely cultivated in a TSB medium at 25 °C for 24 h.

### 2.2 Biofilm Assays

#### 2.2.1 Biomass Production and Inhibition

The quantification of biofilm production of *P. psychrophila* M33T02.2 TH-adapted and nonadapted strains was performed as described by Caballero Gómez et al. (2016).^14^ Briefly, the wells of sterile 12-well polystyrene microtiter plates (TPP, Switzerland) were filled with 2 mL of TSB broth. Overnight bacterial cultures were diluted 1/10 (v/v) in fresh TSB, 200 μL were added to each well, and plates were incubated aerobically for 24 h at 25 °C. To quantify the biofilm formation, the wells were gently washed three times with 2 mL of phosphate-buffered saline (PBS). The adhered bacteria were fixed with 2 mL of methanol (PanReac) for 15 min, and then the microplates were emptied and dried at room temperature. Subsequently, 2 mL of a 2% (v/v) crystal violet solution were added to each well and kept at room temperature for 5 min. Excess stain was removed by placing the plate under gently running tap water. Stain was released from adherent cells with 2 mL of 33% (v/v) glacial acetic acid. The optical density (OD) of each well was measured at 580 nm by using a plate reader (Microplate Tecan). Each assay was performed in triplicate. TSB served as a negative control. The cutoff (OD_C_) was defined as the mean OD value of the negative control. Based on the OD, strains were classified as nonbiofilm producers (OD ≤ OD_C_), weak (OD_C_ < OD ≤ 2 × ODc), moderate (2 × OD_C_ < OD ≤ 4 × OD_C_), or strong biofilm producers (4 × OD_C_ < OD) according to Borges et al. (2012).^15^

The percentage of inhibition of biofilm formation of TH-adapted *P. psychrophila* M33T02.2 with respect to the nonadapted strain was determined using the following formula as described by Zmantar et al. (2012)^16^ in the presence and absence of TH exposure (1/2 MIC).

$$\frac{OD\left(\mathrm{Wild}\;\mathrm{strain}\right)-OD\left(\mathrm{TH}\;\mathrm{adapted}\;\mathrm{strain}\right)}{OD\left(\mathrm{Wild}\;\mathrm{strain}\right)}\times 100$$

#### 2.2.2 Virulence Factors

##### 2.2.2.1 Swarming Motility

The swarming motility of TH-adapted and nonadapted *P. psychrophila* M33T02.2 strains was tested in the presence (150μg/mL) and absence of thymol. Overnight-grown cultures of TH-adapted and nonadapted *P. psychrophila* M33T02.2 strains were diluted to 0.5 McFarland turbidity units and 2 μL spotted on a plate containing swarming medium (Luria Broth (LB) with 1% (v/v) glucose and 0.5% (w/v) agar) and supplemented with 150 μg/mL of thymol or without this. The plates were incubated at 25 °C for 24 h, and swarming zones were observed.^17^

##### 2.2.2.2 Rhamnolipid Production

A rhamnolipid quantification assay was performed according to Lima et al. (2025).^17^ TH-adapted and nonadapted *P. psychrophila* M33T02.2 were grown in 5 mL of LB medium (0.5 McFarland turbidity units) supplemented with 150 μg/mL of TH or without this. Supernatants were collected by centrifugation at 10,000 × g for 10 min and acidified to pH 2 (with HCl) to precipitate rhamnolipids, and absorbance was measured at 570 nm.^18,19^

### 2.3 Confocal Microscopic Evaluation

Biofilms of the TH-adapted and nonadapted *P. psychrophila* M33T02.2 strains were prepared on glass slides by immersion for subsequent imaging. Overnight cultures of both strains were inoculated (1:10 v/v) into 15 mL of Mueller Hinton Broth II (MHBII) at an inoculum density of 0.5 McFarland. The cultures contained glass slides and were supplemented with or without the 1/2 MIC of TH. After 24 h of incubation at 25 °C, three replicates of each sample were washed with sterile distilled water. Staining was performed directly on the slides using 0.5 μL of the LIVE/DEAD BacLight stain (Thermo Fisher Scientific, Waltham, MA, USA). Imaging was conducted using Confocal Laser Scanning Microscopy (CLSM) (LEICA TCS-SP5, Mannheim, Germany), equipped with a Plan-Apochromat 63×/1.4 objective.

### 2.4 Metabolomic Study of TH-Adapted *Pseudomonas psychrophila* M33T02.2 versus Noninduced Strain under 1/2 MIC of TH Treatment

To study the metabolic effects of TH exposure under sub-MIC concentrations on TH-adapted and nonadapted *P. psychrophila* M33T02.2, UHPLC-Q-Orbitrap-MS/MS was used. Overnight cultures of both strains were inoculated following the protocol described above. Next, three replicates of each sample were centrifugated 15 min at 15000 rpm, and the corresponding pellets were stored at −80 °C until analysis.

#### 2.4.1 Metabolite Extraction

Prior to metabolite extraction, the pellets were lyophilized using a Telstar Cryodos 50 freeze dryer (Telstar, Terrassa, Spain). Approximately 2 mg of the lyophilized pellets were aliquoted into 2 mL Eppendorf tubes and resuspended in 500 μL of cold methanol (−80 °C, stored on dry ice). Samples were shaken at 4 °C for 10 min at 950 rpm and subjected to ultrasonic treatment for 10 min at 4 °C^20^ (JP Selecta 3000512, Barcelona, Spain). After briefly vortexing, the addition of 500 μL of ultrapure water, and centrifugation at 14000 rpm for 10 min at 4 °C, 400 μL of the upper phase were placed in 15 mL Eppendorf tubes and vacuum dried in a SpeedVac vacuum concentrator (Eppendorf 5305 Plus, Hamburg, DEU). The dried extracts were fully dissolved in 200 μL of a methanol:water solution prior to analysis (1:1, v/v) and transferred into UHPLC vials (Verex filter vials, 0.2 μm, RC, PTFE/Silicone Pre-Slit).

#### 2.4.2 Polar and Semi-Polar Metabolite Measurements

Metabolites in bacterial extracts were analyzed by UHPLC-MS/MS.^21^ Chromatographic separation was performed using an Ultra High-Performance Liquid Chromatography system (Thermo Fisher Scientific) using a C18 reverse-phase column (100 × 2.1 mm i.d., 1.9 μm particle size, Thermo Fisher Scientific) which operated at a temperature of 40 °C. The mobile phases consisted of 0.1% formic acid in water and 0.1% formic acid in acetonitrile. The flow rate of the mobile phase was 400 μL/min, and 10 μL of sample was loaded per injection. The UHPLC was connected to a high-resolution, accurate-mass (HRMS) spectrometer (Orbitrap-MS/MS). The spectra were acquired by alternating between the full-scan mode without ion fragmentation and the full-scan mode with all-ion fragmentation, in both positive and negative ionization, covering a mass range from 100 to 1200 m/z using the Orbitrap Exploris 120 Mass Spectrometer (Thermo-Fisher, Bremen, Germany). The resolution was set to 60,000, and the maximum scan time was set to 100 ms. All samples were randomized prior to mass spectrometric analysis to avoid any experimental drift.

#### 2.4.3 Metabolite Annotation and Statistical Analysis

The workflow included baseline correction, removal of chemical noise, and chromatogram alignment.^22^ As output, a list of molecular features, that is, a retention time and an m/z ratio pair, and a data matrix containing relative intensities for each feature and for each chromatogram were obtained. All further computations, data manipulations, and plot generation were carried out using the R programming language (http://www.r-project.org). Molecular features were putatively annotated by searching the m/z value against online databases (KEGG, Metabolika, ChemSpider compound databases). A maximum tolerance of 5 ppm was allowed, considering the following potential known adducts: [2M+H]^+1^, [M–H]^−1^, [M+FA–H]^−1^, [M+H–H_2_O]^+1^, [M+H]^+1^, [M+H+MeOH]^+1^, and [2M–H]^−1^ for positive and negative modes. The MS/MS fragmentation of the metabolites was compared against a predicted composition formula and candidate molecules found in the databases. After filtering, the feature data were normalized by the sample dry weight and sample batch median intensity. Due to machine sensitivity variation across different measurement runs, each feature was normalized by dividing its intensity in a sample by the median intensity across all measurements per batch in order to compensate for said effect. Sparse partial least-squares (sPLS) and sparse partial least-squares discriminant analysis (sPLS-DA) were performed using the package mixOmics. In both cases, the metabolite levels were considered to be predictors. For each response variable (categorical or quantitative), a single sPLS/sPLS-DA model was established. To determine the optimal number of components and variables of a given model, we searched the parameter space spanned by 1–12 (3 for sPLS-DA). For each such component/variable combination, 1,000 iterations of 3- to 5-fold cross-validation rounds were tested and the pair that resulted in the lowest (sPLS-DA) or highest R2 (sPLS) classification error was taken as the optimal parameters. Once an optimal number of components and variables was determined for each response variable, we computed the respective sPLS/sPLS-DA model, and using this, we obtained the variable importance in projection (VIP) coefficient for each metabolite.

### 2.5 Statistical Analysis

Statistical analyses were performed using Excel 2016 (Microsoft Corporation, Redmond, WA, United States) to determine averages and standard deviations. A Student’s *t* test was performed at the 95% confidence interval in order to determine the statistical significance of data in biofilm assays (biomass production and virulence factors). All analyses were performed in triplicate.

## 3 Results

### 3.1 Effect of Thymol on *P. psychrophila* M33T02.2 Biofilm Characteristics

#### 3.1.1 Effects on Biomass Production

First, we compared the ability of TH-adapted and nonadapted (WT) *P. psychrophila* M33T02.2 strains to produce biofilms. The results showed that both strains were strong biofilm producers (Figure 1A,B). However, statistical analysis revealed significant differences, showing a lower biomass production for the TH-adapted *P. psychrophila* M33T02.2 strain under both conditions (exposure or not to 1/2 MIC of TH) (Figure 1A). In addition, when strains were exposed to the 1/2 MIC of TH, adapted *P. psychrophila* M33T02.2 decreased its biofilm-forming capacity from strong to moderate (Figure 1A). Interestingly, as shown in Figure 1C, the biofilm formed on the microtiter plate showed clear differences between both strains (TH-adapted and nonadapated strain “WT”), forming a disaggregated biofilm at the air–liquid interface in the case of TH-adapted *P. psychrophila* M33T02.2 strain.

**Figure 1:**
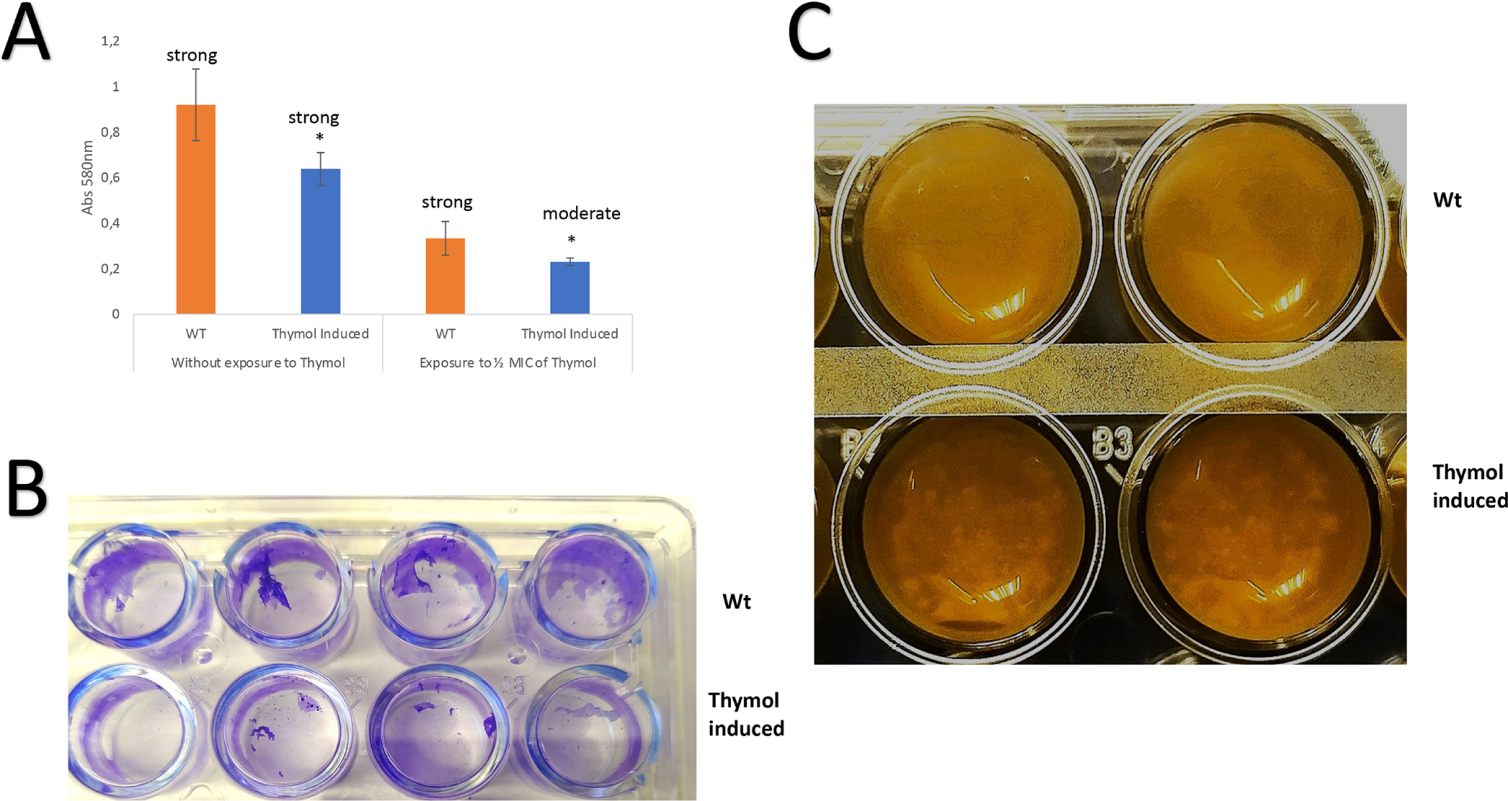
Effect of thymol on biofilm formation in TH-induced and noninduced (wild strain) *Pseudomonas psychrophila* M33T02.2 strains. **A.** Biofilm formation was done using the microtiter plate assay. Data are mean ± SD. The cutoff (OD_C_) was defined as the mean OD value of the negative control. Based on the OD, strains were classified as nonbiofilm producers (OD_C_ ≤ ODC), weak (OD_C_ < OD ≤ 2 × OD_C_), moderate (2 × OD_C_ < OD ≤ 4 × OD_C_), or strong biofilm producers (4 × OD_C_ < OD). **B.** Observation *in situ* of biofilms stained with crystal violet. **C.** Observation *in situ* of differences in the growth patterns. *Indicates a statistically significant biomass reduction with respect to the wild type strain.

#### 3.1.2 Effects on Biofilm Structure

Confocal laser scanning microscopy (CLSM) revealed that biofilms were dense in the nonadapted (WT) *P. psychrophila* M33T02.2 strain. Green fluorescence indicated the presence of viable cells, and red fluorescence indicated the presence of dead cells (Figure 2). When comparing TH-adapted and nonadapted *P. psychrophila* M33T02.2 strains, the surface structure of the biofilm was evidently more discontinuous, showing a sparse dispersion (Figure 2A,C) in the absence of 1/2 MIC of TH. This suggests that TH adaptation had a structural impact on the biofilm architecture of bacteria, resulting in less homogeneous biofilms. On the other hand, the TH-adapted strain exposed to the 1/2 MIC of TH showed a more severe damage to biofilm integrity and a higher mortality of bacteria, as could be seen when measuring a cut on the *Z*-axis (Figure 2D). This was consistent with the results of the biofilm inhibition rate observed for TH-adapted *P. psychrophila* M33T02.2.

**Figure 2:**
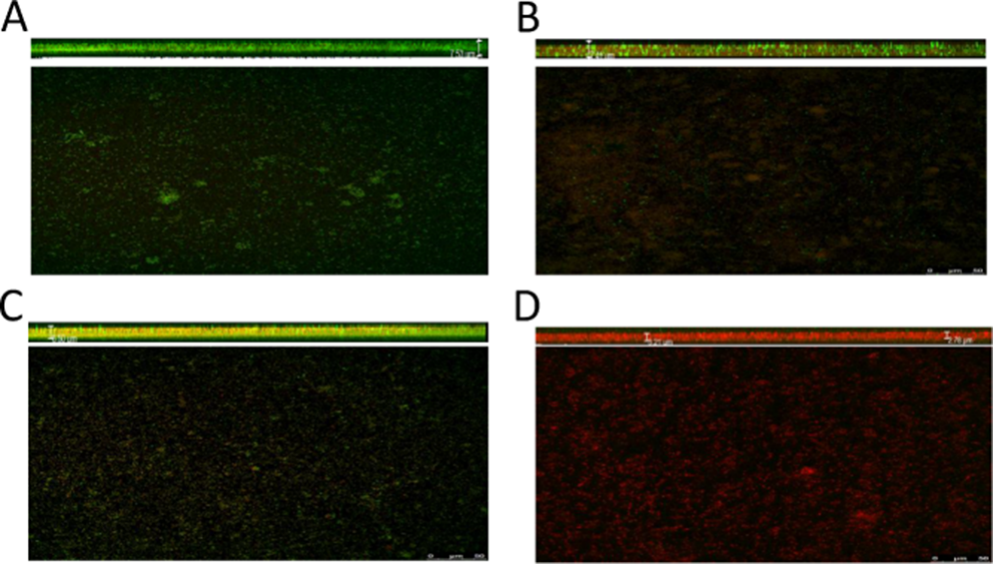
Confocal laser scanning microscopy images of TH-induced and noninduced *Pseudomonas psychrophila* M33T02.2 strains. Live/Dead staining of noninduced (**A, B**) and TH-induced (**C, D**) in the absence (**A, C**) and presence (**B, D**) of 1/2 MIC of TH.

### 3.2 Effects on Virulence Factors

When analyzing the production of rhamnolipids, we observed that in both cases, the production was lower for TH-adapted *P. psychrophila* M33T02.2. The adapted strain had approximately 34.64% less rhamnolipid production under standard conditions, while under 1/2 MIC of TH exposure, 28.4% less production was observed (Figure 3A).

**Figure 3:**
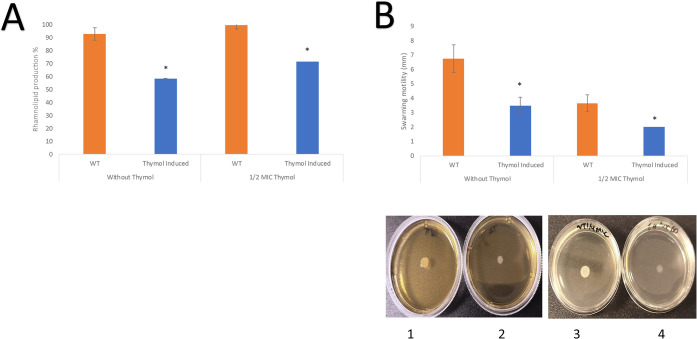
Effect of thymol on virulence factors in TH-induced and noninduced (wild-type strain) *Pseudomonas psychrophila* M33T02.2 strains in the presence and absence of 1/2 MIC of thymol. **A.** Rhamnolipid production. **B.** Swarming motility. Data are means (*n* = 3) ± SD. *Significant differences according to *t* test (*p* < 0.05). Photographs corresponding to swarming motility assays. 1 and 2, Wild and thymol-induced strains without thymol, respectively. 3 and 4, Wild-type and thymol-induced strains in the presence of 1/2 MIC of thymol.

On the other hand, swarming motility tests also showed clear differences between TH-adapted and nonadapted strains. The swarming motility of TH-adapted *P. psychrophila* M33T02.2 was significantly inhibited (Figure 3B), approximately 50% in the absence of TH, as well as the growth observed under 1/2 MIC exposure of TH was very weak and of low density (Figure 3B).

### 3.3 UHPLC-Q-Orbitrap-MS/MS Metabolomic Analysis

We then investigated the effect of TH induction under 1/2 MIC of TH exposure by UHPLC-Q-Orbitrap-MS/MS. 51 polar and semi-polar metabolites were annotated as putative (Table S1, Supplementary Materials). Sparse partial least-squares (sPLS) and sparse partial least-squares discriminant analysis (sPLS-DA) were performed to better understand the significance of these metabolites into the TH adaptation metabolic response. The sPLS-DA components were obtained and plotted (Figure 4A), and the importance of the different metabolite matrices was demonstrated. There was a clear separation between both groups, indicating metabolic differences between the wild-type (nonadapted) and the TH-adapted *P. psychrophila* M33T02.2 strains, and this separation was mainly due to component one, which corresponds to the wild-type strain. Graphical PLS-DA loading of metabolites in the first component of the model was also obtained (Figure S1, Supplementary Materials). Furthermore, in order to identify significantly different metabolites between the two groups, the variable importance in projection (VIP) coefficient was obtained for each metabolite, with those having values over 1 represented (Figure 4B). All metabolites with high significance in the model separation were derived from the wild-type condition. These metabolites were mainly related to redox and energetic metabolism.

**Figure 4:**
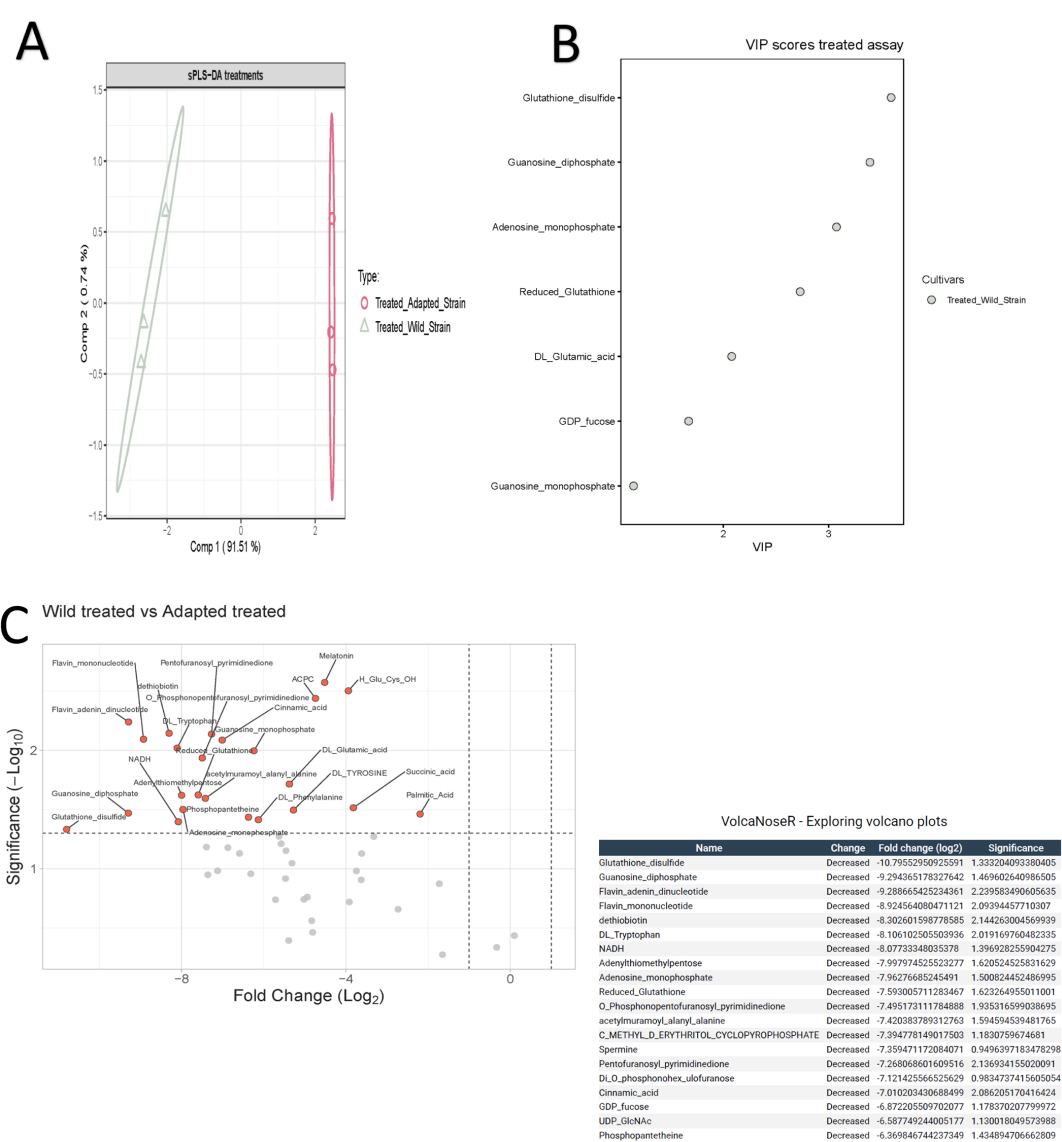
UHPLC-Q-Orbitrap-MS/MS analysis of TH-induced and noninduced *Pseudomonas psychrophila* M33T02.2 strains exposed to 1/2 MIC of thymol. **A.** PLS-DA plot: the pink dots and the gray triangles represent TH-induced and noninduced *P. psychrophila* M33T02.2 strains, respectively, both treated with 1/2 MIC of thymol (75 μg/mL). **B.** Variable importance in the projection (VIP) coefficient. **C.** Volcano plot of metabolites: the abscissa is the FC value of log2, and the ordinate is the *p*-value of −log10, which makes the gap between the substances with great differences large and the substances with small differences narrow.

Metabolic differences between nonadapted and TH-adapted *P. psychrophila* M33T02.2 strains under 1/2 MIC of TH exposure were confirmed using one-step analysis. The *t* test was used to determine the differences between the two groups. The calculation of the metabolite abundance ratio between the two groups, the difference multiple, or fold change value (FC value) was obtained. By combining the *p*-value and FC value for each metabolite, a volcano map of metabolites was drawn (Figure 4C,D). The results showed that there are significant differences in metabolites after 1/2 MIC of TH exposure, between nonadapted and TH-adapted strains, with 20 metabolites underexpressed for the TH-adapted strain (FC log2 value greater than 6), accounting for 39% of the total identified metabolites, including the presence of glutathione disulfide, guanosine diphosphate, and flavin adenine mono- and dinucleotide, among others (Figure 4C).

In summary, 51 metabolites were selected for the implementation of hierarchical Pearson clustering (Figure 5), revealing a statistically significant discrepancy between the wild-type and the TH-adapted *P. psychrophila* M33T02.2 strains. This finding was consistent with the outcomes of the PLS-DA analysis. In particular, redox metabolism intermediates (glutathione disulfide, flavin mononucleotide, and flavin adenine dinucleotide) and energetic balance intermediates (adenosine monophosphate and guanosine diphosphate) stood out as underproduced metabolites with respect to the control with fold changes over 10 (Figure 5), corresponding to VIP values over 1 in the PLS-DA model.

**Figure 5:**
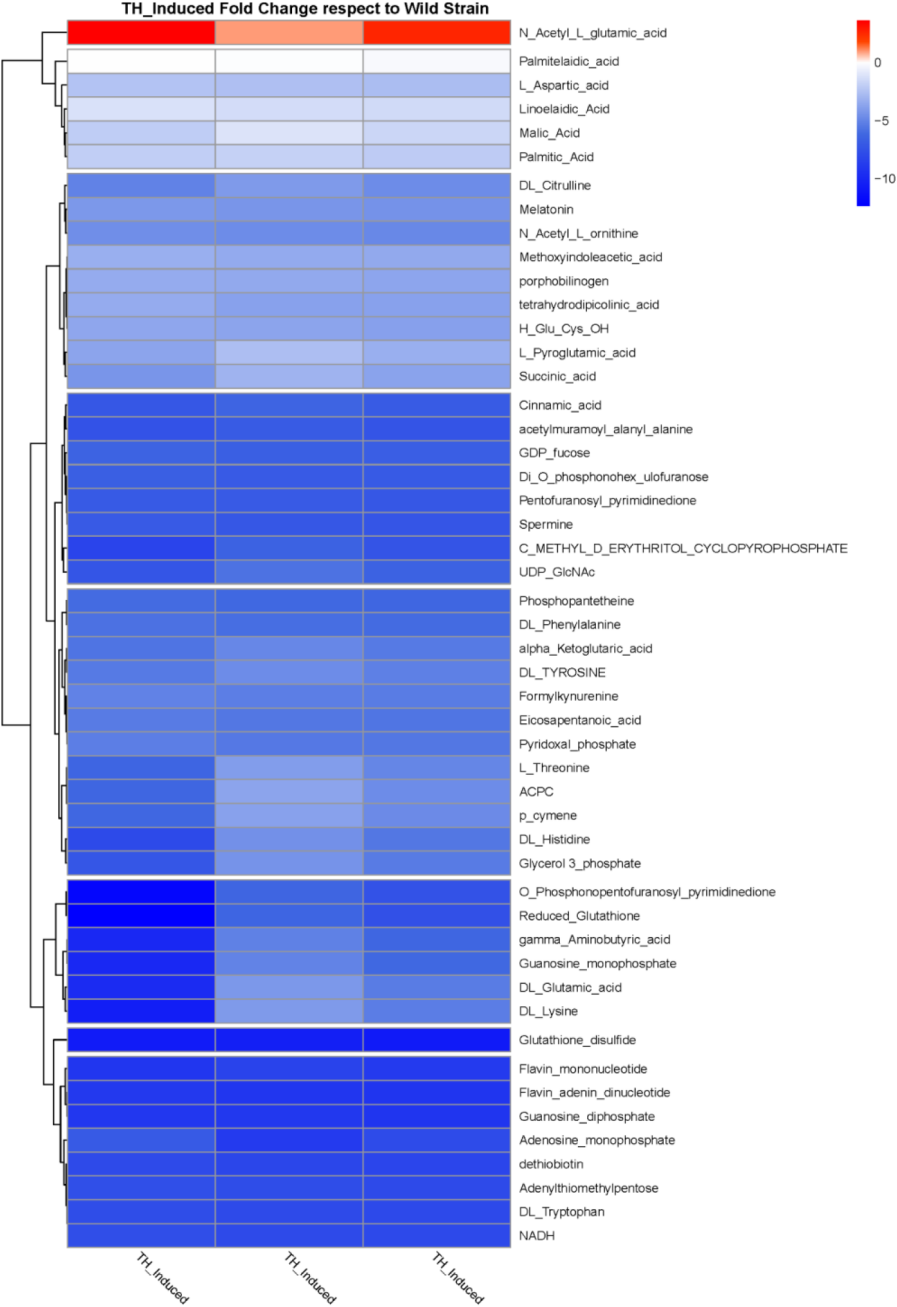
Heatmap and differential metabolite screening, generated by hierarchical Pearson clustering.

## 4 Discussion

Essential oil components, such as thymol, carvacrol, and cinnamaldehyde, have shown promising potential to control multiresistant *Pseudomonas* spp., both in their planktonic and biofilm states.^12,23,24^ Our previous work^12^ demonstrated that TH is highly effective against *P. psychrophila* M33T02.2 in both planktonic and sessile states. However, it is crucial to understand the effects of TH exposure on adapted strains before establishing a new disinfection strategy, thereby simulating a real-world scenario following successive adaptation to TH-based disinfectants.

Biofilms are associated with resilience and make infections difficult to treat in many bacteria.^25^ Therefore, investigating and assessing disinfectant strategies aimed at reducing the biofilm-forming capacity of pathogenic bacteria linked to food environments are currently key objectives for food safety. It is essential to find new, efficient, and sustainable strategies to limit antimicrobial resistance (AMR) spread and the increasing occurrence of biofilm-associated infections.^26^

We therefore studied the effects of TH adaptation of *P. psychrophila* M33T02.2 on the biofilm formation. To do so, we first evaluated the impact on biofilm abundance when *P. psychrophila* M33T02.2 was adapted to increasing sublethal concentrations of TH. Our results demonstrate that TH adaptation of *P. psychrophila* M33T02.2 resulted in a 33% decrease in biofilm production compared to the wild type (nonadapted), in both situations studied (with and without the presence of 1/2 MIC of TH). In line with this, Strantzali et al.^27^ and Čabarkapa et al.^28^ found that the application of TH at 1/2 MIC caused a significant inhibition of *Salmonella* biofilm formation.

Biofilm formation is a complex process involving many factors, notably bacterial swarming motility and exopolysaccharides.^29^ It is well known that swarming motility is a result of a complex multicellular process involving flagella.^30,31^ In this regard, in our previous study (Caballero Gómez et al., under review), we detected four underexpressed genes related to flagella function (*fliM*, *fliE_2*, *fliG_2*, and *flgB*) in TH-induced *P. psychrophila* M33T02.2. These virulence factors facilitate sensing or searching for a suitable surface and finally progressing from reversible to irreversible attachment.^32^ The TH-induced *P. psychrophila* M33T02.2 strain showed a 50% reduction in swarming motility compared with the noninduced strain, both with and without 1/2 MIC of TH. This finding could explain the lower rate of biofilm formation observed. Additionally, exopolysaccharide production affects the swarming motility of *P. aeruginosa* by controlling rhamnolipid production^29^ due to their surfactant properties. Our study shows that rhamnolipid production is reduced by 30% after prolonged exposure to increasing concentrations of TH and remains reduced once the strain has been adapted. Thus, we can conclude that prolonged exposure to subinhibitory concentrations of TH affects the ability to form biofilm. These data were supported by confocal microscopy, showing that biofilms developed by the TH-adapted strain were less homogeneous, and treatment with sub-MIC of TH had the greatest effect on the biofilms developed by the adapted strain to this component. In this context, our results are consistent with previous studies. Ahmed et al.^33^ showed that *trans*-cinnamaldehyde (CA) and salicylic acid (SA) significantly inhibited the expression of QS regulatory and virulence genes in *P. aeruginosa* PAO1 at subinhibitory levels. *In silico* molecular docking studies revealed that TH interacts with the QS receptor CviR in *Chromobacterium violaceum*,^34^ and Naik et al.^35^ demonstrated the action of EOCs, including TH, as QS inhibitors.

To further understand the effect of TH adaptation on resistance development, we conducted a metabolomic study comparing the effects of 1/2 MIC of TH exposure on the TH-adapted and nonadapted *P. psychrophila* M33T02.2 strains. PLS-DA results derived from UHPLC-Orbitrap-MS/MS metabolites yielded redox metabolism intermediates (glutathione disulfide, flavin mononucleotide, flavin adenine dinucleotide) and energetic balance intermediates (adenosine monophosphate, guanosine diphosphate, among others) as VIPs after TH adaptation of *P. psychrophila* M33T02.2 and as underproduced metabolites with respect to the control with fold changes over 10. It is well known that metabolome profiling has been mainly used to characterize enzyme inhibitors, since the inhibition of a metabolic enzyme results in increased levels of its substrates and decreased levels of its products.^36^ Our results showed large differences in the presence of secondary intermediates of metabolic importance such as GDP and GMP, showing underexpression values of 9 for GDP. High c-di-GMP levels are associated with increased matrix production and biofilm structure formation, while low c-di-GMP levels are associated with planktonic growth and/or biofilm dispersion.^21^ The levels of c-di-GMP in the cell are modified by the rate of its synthesis and degradation. The molecule of c-di-GMP is synthesized from two molecules of GTP by enzymes called diguanylate cyclases (DGCs) and is degraded into 5-phosphoguanylyl-(3′,5′)-guanosine (pGpG) and/or GMP by phosphodiesterases (PDEs).^37^ Interestingly, FAD appear underexpressed (FC 2log −8,9). In this regard, Rossi et al.^25^ suggested that FAD may exert a fine-tuning of the GTP-dependent PDE in response to the cellular redox state regulating protein function in a redox-dependent manner.^38^ As an example, we can observe how the growth of biofilms in nutrient sources is characterized by an excess of intracellular electron donors and a shortage of electron acceptors.^39^

Finally, our metabolomic results show that when the TH-adapted *P. psychrophila* M33T02.2 is re-exposed to sublethal (1/2 MIC) TH concentrations, an energy and redox imbalance appear, directly affecting pathways related to biofilm formation. These results are consistent with previous data, which showed lower biofilm formation capacity, as well as lower swarming and rhamnolipid production. Similar results were obtained by Pejčić et al.^40^ who confirmed the effect of EOCs not only on the inhibition of biofilm formation but also on the reduction of swimming, swarming, and twitching motility patterns in *Pseudomonas aeruginosa* clinical isolates.

Additionally, other studies have highlighted the antimicrobial and antioxidant roles of thymol.^41^ The adaptive bacterial response to thymol has also been reported, for example, in *E. coli* O157:H7, resulting in enhanced resistance against subsequent lethal EOCs, heat, and oxidative stresses. In this context, Yuan et al.^42^ revealed through a transcriptomic analysis the upregulation of stress resistance genes and a downregulation of various virulence genes in EOC-adapted cells. Furthermore, Di Pasqua et al.^43^ reported that thymol adaptation plays a role in altering very different pathways of cell metabolism in *Salmonella enterica* serovar Thompson.

We can conclude that repeated exposure to TH not only prevents the emergence of resistance mechanisms associated with biofilm formation but also weakens the bacteria’s ability to form biofilms that promote a resistant state. This suggests that impeding biofilm formation and the resulting antimicrobial resistance through repeated TH exposure, particularly given its effects on the redox state and energy imbalance, present an interesting avenue for exploration. Further studies will be necessary to fully confirm these interactions and mechanisms.

## Supplementary Material



## Notes

(Figure S1) PLS-DA loadings of metabolites; (Table S1) The list of significant differential metabolites detected by UHPLC-Q-Orbitrap-MS/MS analysis in *Pseudomonas psychrophila* M33T02.2 versus the noninduced strain under 1/2 MIC of TH treatment (PDF)
