# Supplementary material for: Pseudomonas psychrophila Biofilm Formation Inhibition by Thymol Adaptation
Source: J Agric Food Chem. 2026 Jan 7;74(2):2315–23. doi: 10.1021/acs.jafc.5c09527 (PMC12833837; doi:10.1021/acs.jafc.5c09527)
Supplement: Supplementary file 1 [file jf5c09527_si_001.pdf]

Supporting Information

Pseudomonas psychrophila biofilm formation inhibition by Thymol adaptation

Natacha Caballero Gómez[1], Julia Manetsberger[1], Carlos Terriente-Palacios[2], José G. Vallarino[2], Nabil Benomar[1], Hikmate Abriouel[1]*

[1]Area of Microbiology, Department of Health Sciences, Faculty of Health sciences, University of Jaén, 23071-Jaén, Spain

[2]Department of Molecular Biology and Biochemistry, Institute of Subtropical and Mediterranean Horticulture 'La Mayora', University of Malaga - Consejo Superior de Investigaciones Científicas (IHSM-UMA-CSIC), 29010-Malaga, Spain

*** Correspondance: hikmate@ujaen.es**

Figure S1. PLS-DA loadings of metabolites.

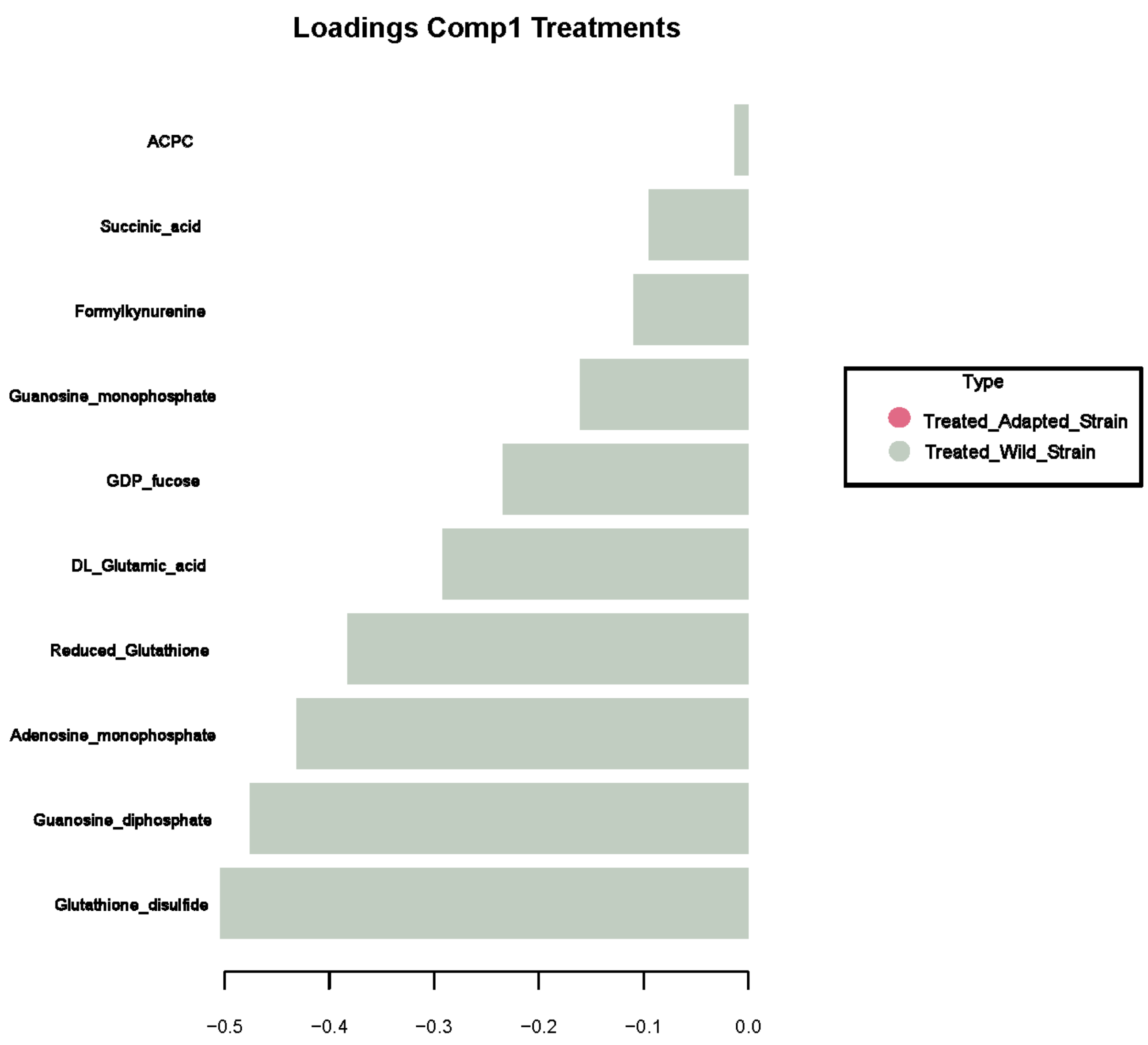
Loadings Comp1 Treatments
ACPC
Succinic_acid
Formylkynurenine
Guanosine_monophosphate
GDP_fucose
DL_Glutamic_acid
Reduced_Glutathione
Adenosine_monophosphate
Guanosine_diphosphate
Glutathione_disulfide
−0.5
−0.4
−0.3
−0.2
−0.1
0.0
Type
Treated_Adapted_Strain
Treated_Wild_Strain

Table S1. The list of significant differential metabolites detected by UHPLC-Q-Orbitrap-MS/MS analysis in Pseudomonas psychrophila M33T02.2 versus non-induced strain under ½ MIC of TH treatment.

Name	Wild type_1	Wild type_2	Wild type_3	TH induced_1	TH induced_2	TH induced_3
(±)-Malic acid	1,661819853	1,329795016	1,312870185	0,353142161	0,670204984	0,449095895
1,6-Di-O-phosphonohex-2-ulofuranose	0,685574856	0,833776331	0,666767268	0,006869675	0,005842228	0,005578618
1-(5-O-Phosphonopentofuranosyl)-2,4(1H,3H)-pyrimidinedione	8,113331797	6,919725938	6,597257387	0,001892711	0,090340772	0,040476664
1-Pentofuranosyl-2,4(1H,3H)-pyrimidinedione	0,640011713	0,573264355	0,532446202	0,004481248	0,00456464	0,003969788
2,3,4,5-tetrahydrodipicolinic acid	0,049247975	0,076963378	0,055387852	0,005537307	0,004296165	0,004315419
2-C-METHYL-D-ERYTHRITOL-2,4-CYCLOPYROPHOSPHATE	2,449562264	1,755815724	1,84553013	0,006324418	0,021604641	0,012256668
4′-Phosphopantetheine	0,416292195	0,324738342	0,325201251	0,005456652	0,004821399	0,004510523
ACPC	4,710961049	4,401724727	3,999102153	0,055377069	0,326976708	0,167795955
Adenosine monophosphate	12,37008504	11,89784059	10,64997916	0,089585196	0,024993797	0,050282991
Adenylthiomethylpentose	7,362624834	5,976813081	5,854012329	0,030896294	0,028968977	0,026271874
alpha-Ketoglutaric acid	0,714346785	0,43319132	0,503597097	0,010712398	0,018396332	0,012774366
Caffeine	0,325878696	0,1971202	0,229518065	0,025356733	0,022752851	0,021112891
Cinnamic acid	39,04117572	35,20154338	32,58141728	0,245803272	0,410124169	0,287853758
dethiobiotin	0,635095047	0,543238208	0,517111549	0,002307816	0,001988196	0,001885305
DL-Citrulline	0,199549772	0,681680892	0,386728077	0,012429306	0,024710766	0,016298921
DL-Glutamic acid	15,67382634	15,24016003	13,56660292	0,017435024	0,810477203	0,363329281
DL-Histidine	1,91296236	1,434839528	1,469182858	0,006417305	0,07137801	0,034140474
DL-Lysine	16,56338876	20,13429983	16,10478064	0,011743581	1,021944088	0,453633834
DL-Phenylalanine	1,488925097	1,155543538	1,16052506	0,022734915	0,02055764	0,018998938
DL-Tryptophan	1,398570479	1,260740463	1,167038607	0,005909761	0,005214693	0,004881967
DL-TYROSINE	0,289825655	0,233258683	0,229555562	0,00582191	0,009499762	0,006723916
Eicosapentanoic acid	0,301165288	0,145199793	0,195887316	0,005125681	0,004496267	0,004222592
Flavin adenin dinucleotide	8,420469265	7,235503532	6,870623662	0,015256327	0,013547884	0,012640728
Flavin mononucleotide	3,107151572	2,791614619	2,588673543	0,005160462	0,008661992	0,006065984
Formylkynurenine	0,31580813	0,343661037	0,289408044	0,009068721	0,008329023	0,007635
gamma-Aminobutyric acid	2,670203971	1,688867064	1,912978324	0,002117967	0,058016035	0,026389807
GDP-fucose	0,237260419	0,255460482	0,216230567	0,002620941	0,002257955	0,002141104
Glutathione disulfide	3,306210213	3,349151285	2,920705393	0,00197902	0,002329916	0,001890976
Glycerol 3-phosphate	1,610622197	0,249869367	0,816476723	0,005863449	0,041459427	0,020767644
Guanosine diphosphate	4,581051485	4,459948181	3,967642703	0,008200238	0,008391841	0,007281434
Guanosine monophosphate	1,629900426	1,508552068	1,377309877	0,001556385	0,044688195	0,020294434
H-γ-Glu-Cys-OH	0,249755745	0,216930446	0,204805235	0,017904829	0,017057799	0,01534335
L-(+)-Aspartic acid	0,190397733	0,088321892	0,122316107	0,02494636	0,022455073	0,020802119
L-(−)-Threonine	0,88073563	1,446486136	1,021301272	0,013503436	0,068383007	0,035935866
L-Pyroglutamic acid	7,784277818	6,34231089	6,199453454	0,504865827	1,092468549	0,700990191
Linoelaidic Acid	0,104424209	0,611546772	0,314203865	0,14784676	0,12869858	0,121361923
Melatonin	0,334336848	0,29023004	0,274091179	0,016287122	0,015112816	0,013779863
Methoxyindoleacetic acid	0,058871778	0,041286398	0,043954415	0,004992927	0,004346759	0,004098721
N-Acetyl-L-glutamic acid	0,124845838	0,118282156	0,10669672	1,401246168	0,224521575	0,713468174
NADH	1,985507861	1,531434762	1,54341027	0,008074526	0,006866878	0,006557035
N~2~-Acetyl-L-ornithine	0,298503927	0,454218221	0,330332115	0,014963774	0,013806751	0,012625945
p-cymene	1,271260289	3,077326792	1,90837744	0,027823997	0,148782145	0,077503605
Palmitelaidic acid	0,059693933	0,077804816	0,060341326	0,064892763	0,062589741	0,055945697
Palmitic Acid	0,303708664	0,30161729	0,265647295	0,071797411	0,080486343	0,066829725
porphobilinogen	0,369228929	0,440208771	0,355221735	0,036155387	0,034061449	0,030814658
Pyridoxal 5′-phosphate	0,648984308	0,481005524	0,495896038	0,014097978	0,01213807	0,01151369
Reduced Glutathione	7,685623051	7,318188782	6,584422823	0,001396224	0,084911118	0,037875977
Spermine	1,089499085	1,363505251	1,076500953	0,009191026	0,008210564	0,007636688
Succinic acid	16,39376397	16,6689747	14,50958287	0,805009692	1,861050147	1,17000036
UDP-GlcNAc	0,951842334	1,062726134	0,884093372	0,005928582	0,017867735	0,010443014
UDP-N-acetylmuramoyl-L-alanyl-γ-D-glutamyl-meso-2,6-diaminopimeloyl-D-alanyl-D-alanine	0,797548111	0,646272786	0,633620801	0,003849303	0,005735522	0,0042063
